# Relationships between proximity to grocery stores and Oklahoma Early Care and Education classroom nutrition practices

**DOI:** 10.1016/j.pmedr.2022.101917

**Published:** 2022-07-21

**Authors:** Bethany D. Williams, Susan B. Sisson, Bryce C. Lowery, Dipti A. Dev, Diane M. Horm, Janis E. Campbell, Denise A. Finneran, Jennifer Graef-Downard

**Affiliations:** a Department of Nutritional Sciences, College of Allied Health, University of Oklahoma Health Sciences Center, 1200 N. Stonewall Ave., Oklahoma City, OK 73114, USA; b Department of Nutrition and Exercise Physiology, Elson S. Floyd College of Medicine, Washington State University Health Sciences Spokane, 412 E Spokane Falls Blvd, Spokane, WA 99202, USA; c Department of Regional and City Planning, Gibbs College of Architecture, University of Oklahoma, 830 Van Vleet Oval, Norman, OK 73019, USA; d Department of Child, Youth and Family Studies, College of Education and Human Sciences, University of Nebraska-Lincoln, Louise Pound Hall, 512 N 12th St, Lincoln, NE 68588, USA; e Early Childhood Education Institute, University of Oklahoma-Tulsa, 4502 E. 41st St., Tulsa, OK 74135, USA; f Department of Biostatistics and Epidemiology, Hudson College of Public Health, University of Oklahoma Health Sciences Center, 801 N.E. 13th Street, Oklahoma City, OK 73104, USA; g Department of Communication Sciences and Disorders, College of Allied Health, University of Oklahoma Health Sciences Center, 1200 N. Stonewall Ave., Oklahoma City, OK 73114, USA

**Keywords:** ECE, Classroom nutrition practices, Barriers, Proximity to grocery stores, GIS

## Abstract

The study purpose was to determine associations between proximity to grocery stores and Early Care and Education programs’ (i.e., ECEs) classroom nutrition practices and barriers, by ECE context (Head Start, community-based childcare [CBC], and family child care homes [FCCHs]). A statewide cross-sectional survey was implemented in Oklahoma ECEs. Directors reported classroom nutrition practices with the Nutrition and Physical Activity Self-Assessment tool, and barriers to implementation. Locations of 457 grocery stores statewide were determined by in-person audit. Geocoded ECEs were considered within a “low proximity” area if no grocery stores were available within a 0.25-mile radius for urban, or 10-mile radius for rural, ECEs. From November 2019 to February 2020, 54 Head Starts, 159 CBCs, and 160 FCCHs participated. 31.0 % were considered as low proximity. Head Starts demonstrated the highest classroom nutrition scores for mealtime practices, and nutrition education and policy. While proximity to grocery stores was not related to classroom nutrition practices for any ECE context (p > 0.05), FCCHs located within a low proximity area reported barriers to implementing those practices more often compared to FCCHs in an area within accessible proximity of grocery store. Thus, proximity to grocery stores was related to barriers in FCCHs only; those provider’s experiences and perceptions may be most susceptible to influence of the community nutrition environment, compared to other ECE contexts. Contrary to studies in residential areas and schools, nutrition environments were not related to nutrition practices in ECEs. ECEs may serve as protective micro-environments supporting health for children residing in nearby low-access communities.

## 1 Introduction

Encouraging healthful diet in early childhood can promote healthy weight maintenance, development, and metabolic function (Lorson et al., 2009, Lin and Morrison, 2002). Independent of childhood weight gain, the importance of meeting nutrient needs in early childhood is well known, with adequate consumption of specific nutrients predicting cognitive and verbal skills (Hubbs-Tait et al., 2009), academic performance (Shariff et al., 2000), and proper growth trajectories for height and bone development (Rivera et al., 2003). Dietary habits developed in youth persist into adulthood (Birch and Fisher, 1998, Mikkila et al., 2005, Mikkila et al., 2004); and therefore influence lifelong health outcomes. For these reasons, federal guidelines recommend that young children ages 2 to 5 years should consume foods that are more nutrient-dense than calorie-dense; such food groups include low-fat milk/dairy, lean meat and beans, fruit, vegetables, and whole grains (USDA, 2019, Dietary Guidelines for Americans, 2015, USDA, 2018, USDA, 2017, Dietary Recommendations for Healthy Children, 2018). However, only 14 % of US children currently consume recommended servings of fruits and vegetables for their age ranges (Kunin-Batson et al., 2015), suggesting a need to understand how to best support healthful diet for young children nationwide.

Current efforts have targeted early childhood education (ECEs) settings to promote a healthful diet in young children. Serving healthful foods and encouraging their consumption in ECEs is promising, as more than 60 % of US children below age five attend ECEs (Laughlin, 2011, Redford et al., 2001). General licensing requirements for ECEs do not enforce nutrition-related requirements or standards for foods served to children. This said, federal programs such as the Child and Adult Care Food Program (CACFP) provide funding to ECEs reimbursing the purchase of healthful food served to young children from families who qualify as low-income (US Dept of Agriculture. Child and Adult Care Food Program, 2017). Studies support the effectiveness of these efforts, reporting that foods served in ECEs are more healthful than those offered in children’s homes (Sisson et al., 2017) and that CACFP-participating ECEs have more healthful classroom environments than do those non-CACFP ECEs (Andreyeva and Henderson, 2018). However, not all ECEs participate in such programs, and there is variable fidelity for meeting required standards among those who do, which vary by ECE context (Head Start, community-based childcare, and family child care homes) (Monsivais et al., 2011, Schwartz et al., 2015). In addition to serving healthful foods, ECEs are recommended to implement best practices at mealtime, including responsive feeding practices and family-style meal service, to promote a social mealtime environment and encourage children to consume the nutrient-dense food items they are served (Benjamin Neelon and Briley, 2011). These mealtime best practices and responsive feeding provide added benefits for young children, including child development of social skills (Harte et al., 2019), fine motor skills (Rule and Stewart, 2002), autonomy (Harte et al., 2019), and preference for nutritious foods (Benjamin Neelon and Briley, 2011, Birch, 1998). For Head Starts, which receive resources for classroom health practice implementation through articulated Program Performance Standards (Head Start Program Performance Standards and Other Regulations), young children’s attendance is related to lowered rates of obesity for those students (Frisvold and Lumeng, 2011). To develop effective intervention it is essential to understand factors influencing ECE implementation of classroom nutrition practices, including for foods served and mealtime best practices.

Across all age groups, individuals living in neighborhoods with limited access to healthy foods consume more energy-dense foods and fewer fresh fruits and vegetables (Drewnowski and Poverty, 2004). For middle- and high-school aged children, food outlets surrounding schools can predict out-of-home food purchasing and dietary consumption (Cutumisu et al., 2017, Williams et al., 2014). There is evidence indicating that community nutrition environments surrounding children’s frequented locations, including the home and school, influence food accessibility and intake (Cutumisu et al., 2017, Williams et al., 2014). However, there are few studies examining the impact of proximity to grocery store outlets surrounding ECEs on those organizations’ health practices, and thus on the young children they serve (Burgoine and Gallis, 2017, Braun et al., 2022). While older children’s autonomous food purchasing decisions are impacted by the healthfulness of nearby food outlets (Bassett et al., 2008, He et al., 2012), younger children may be more likely to be influenced by how the ECE itself responds to being located within a low-proximity area.

Access-related barriers to implementing classroom nutrition practices, including limited space for storing foods and lack of resources within the ECE program (Gunter et al., 2012, Hughes et al., 2010, Lindsay et al., 2015, Tovar et al., 2015, Zaltz et al., 2018, Dev et al., 2014), have been identified. Understanding how the surrounding community is related to ECE classroom practices is essential to inform health-impacting programs, policies, and community resources. The primary purpose of this study was to determine associations between ECE proximity to grocery stores with classroom nutrition practices and barriers, by ECE context (Head Starts, community-based childcare [CBCs], and family child care homes [FCCHs]). Findings from residential studies report that children living in areas with low access to healthful food outlets have lower diet quality and higher preference for energy-dense foods and beverages (Cutumisu et al., 2017, Williams et al., 2014). Thus, we hypothesized that ECEs located within an area with low proximity to grocery stores would similarly demonstrate less healthy classroom nutrition practice scores across various practice types, including quality of foods/beverages served in the classroom and on menus, and active promotion of healthy foods and eating practices. Relatedly, we hypothesized that staff from ECEs with low proximity to grocery stores would report higher barriers which would theoretically impact successful implementation of those classroom nutrition practices, including those related to lack of resources/time, child/family food preferences, and staff member self-efficacy. Previous research in CBCs and FCCHs indicate that FCCHs are more likely to purchase foods in person, and further that online food purchasing may not be cost-efficient or plausible for FCCHs and small CBCs (Lazarus et al., 2018). Thus, we also hypothesized that Head Starts and CACFP-participating CBCs and FCCHs with additional support and resources would be less vulnerable to the influence of the surrounding community environment.

## 2 Materials and methods

### 2.1 Study design, sampling methods, and recruitment strategies.

The Communities and Classroom Health Survey was a cross-sectional study with primary aims to determine associations between health of community environments surrounding ECEs with classroom health and staff-reported barriers; detailed study methods have been reported previously (Williams et al., 2021). In brief, the survey was distributed to all licensed ECEs in the state of Oklahoma from November 2019 to February 2020 (*N* = 2,962). Surveys could be completed on paper or via an online survey link using the Research Electronic Data Capture (REDCap) secure system (Harris et al., 2009). A total of 470 surveys (23.5 % response rate) were received including 64 Head Starts, 207 CBCs, 189 FCCHs, and 10 considered “Other” or ineligible.

This study was conducted according to the guidelines laid down in the Declaration of Helsinki and all procedures involving research study participants were approved by the Institutional Review Board at the University of Oklahoma Health Sciences Center (IRB no. 11083). Need for written informed consent was waived by the IRB, due to data being program-level, not individual-level, and thus was not considered as human subjects research. Consent was assumed by completion of the survey.

### 2.2 Survey instrument and sample characteristics

Surveys included questions regarding ECE location, demographics and food service- related characteristics; specific items assessed ECE hours of operation, number and education of teachers, number and approximate race/ethnicity of attending children, professional development participation, and food preparation/purchasing methods. ECEs reported how food and beverages for the center’s meal service are primarily obtained, and if applicable, number of roundtrip miles to get to the location where those foods are purchased. Classroom nutrition practices and barriers to implementation of nutrition practices were also assessed. Surveys were completed by center directors who were advised to respond to all items in regards to current classrooms serving 3-to-5-year-old children. Directors could ask additional staff for help if they were unsure of how to respond. Items on demographics were derived from a previous statewide survey of Nebraska ECEs (Garcia et al., 2018); variables were reported at the level of the ECE, including information on ECE context (Head Start, CBC or FCCH).

### 2.3 ECE proximity to grocery stores

Healthfulness of the community food environment surrounding participating ECEs was determined in ArcMAP 10.6, and assessed as whether or not the ECE was located within a specified proximity to all grocery stores located across the state of Oklahoma, or within 0.25 miles of the state border. From June 2015 to June 2016, locations of 457 grocery stores throughout the state of Oklahoma were identified and confirmed by in-person store audits. Rural/urban status was mapped and determined each ECE location using census tract-level 2010 Rural-Urban Commuting Area (RUCA) codes (USDA Economic Research Service, 2016) which are assigned based that census tract population density, urbanization, and daily commuting.

Participating ECEs were considered located within a “low proximity” area if no grocery stores were available within an accessible Euclidian distance of 0.25-miles for urban ECEs, or 10-miles for rural ECEs (Moore and Diez Roux, 2006, Centers for Disease Control and Prevention, 2018). These cutoff points have been commonly used in previous literature studying impact of food environments on children’s home and school environments (Williams et al., 2014). To examine validity of this metric and provide context to the current sample and study findings, differences in food purchasing methods, miles to purchasing and nearest grocery stores, and percent urban/rural among by GIS-determined proximity to grocery stores are presented as a Supplementary Material (Supplementary Table 1). In brief, ECEs located within a low proximity area were more likely to purchase foods online then picked up in person, or over the phone with a vendor, compared to their counterparts within accessible proximity of a grocery store. Further, those defined as being withing a low proximity area reported higher miles to purchasing center foods and were more likely to be located in an urban area.

### 2.4 Classroom nutrition practices and barriers.

Classroom nutrition practices were determined using 36 survey items from the full 54-item Nutrition and Physical Activity Self-Assessment tool (i.e., NAPSACC); this survey instrument has been previously validated and is currently published and available (Benjamin et al., 2007). Items were answered on a Likert-type scale from one to four, with higher scores indicating higher frequency or healthier degree of implementing nutrition requirements and best practices. Individual item scores were averaged to create nine sub-section scores. Sub-section scores ranged from one to four, with four being the healthiest. All nine sub-section score averages were then summed to calculate a NAPSACC Nutrition Total Score, which ranged from nine to 36.

Barriers to implementing classroom nutrition practices were determined by 25 items drawn from a previous statewide survey in Nebraska ECEs (Dev et al., 2020). Specifically, there were 14 items to determine barriers to serving healthier food and beverages, and 11 items to determine barriers to employing healthful mealtime best practices. Examples of healthful mealtime best practices were specified on the survey, and included praising children for trying new foods, talking with children about healthy foods, allowing children to decide when they are full, sitting with children during mealtime and eating the same foods, and serving meals family style. Providers were asked whether their ECE experienced each potential barrier (“yes” or “no”).

### 2.5 Statistical analysis

SAS v. 9.4 was used to calculate descriptive statistics (means, standard deviations, and frequencies) and all primary analyses (SAS Institute Inc. 2013, Carey, NC). To account for inherent differences among each ECE context, all primary analyses were performed separately within Head Starts, CBCs, and FCCHs. The Shapiro-Wilk test for normality indicated that primary outcome data were not normally distributed (*p* < 0.05 for all ECE contexts). Kruskal-Wallis one-way analysis of variance was used to determine differences in ECE continuous demographic characteristics and classroom nutrition practice scores between the three ECE contexts (Head Starts, CBCs, and FCCHs). Fisher’s Exact test was also used to determine differences in categorical demographic characteristics and frequency of reporting barriers (percent “yes”) to implementing ECE classroom nutrition practices between the three ECE contexts (Head Starts, CBCs, and FCCHs).

Addressing primary aims of the present study, Wilcoxon Rank Sum test was used to determine differences in ECE classroom nutrition practice scores between those located within low proximity areas versus those within accessible proximity of grocery store, stratified by ECE context. Fisher’s Exact test was used to determine differences in frequency of reporting barriers (percent “yes”) to implementing ECE classroom nutrition practices between those located within low proximity areas versus those within accessible proximity of grocery store, stratified by context. Exploratory analyses were then conducted to further stratify by CACFP participation among CBCs and FCCHs; Head Starts were not included in these additional analyses, since those in this sample almost unanimously participated in CACFP (98.5 %). The Benjamini Hochberg correction was applied to primary analyses to account for multiple comparison and control for False Discovery Rate, with adjusted alpha *p* < 0.004.

## 3 Results

A total of 474 Oklahoma ECEs responded to the survey [33.5 %, 18.2 % and 11.6 % response rate for Head Starts (*n* = 64), CBCs (*n* = 206), and FCCHs (*n* = 192) respectively]. ECEs were excluded if they were a “Public Pre-K” or missing information on ECE context (*n* = 15; 3.1 %). ECEs were further excluded for having missing data on primary variables of interest (*n* = 89; 18.7 %). Sample characteristics of the final analytic sample (373 ECEs, including 54 Head Starts, 159 CBCs, and 160 FCCHs) are described in Table 1.

**Table 1:** Oklahoma Early Care and Education programs participating in the Communities and Classroom Health Survey in 2019–2020, by context (*n* = 373).

	**Head Start** **(*n* = 54)**		**CBC** **(*n* = 159)**		**FCCH** **(*n* = 160)**		
	% or mean	*SD*	% or mean	*SD*	% or mean	*SD*	*p*-value
*Center Hours (%)							
Half Day	31.4	–	6.9	–	3.7	–	<0.0001*
Full Day	81.4	–	94.3	–	96.8	–	*0.0010
“Other”	0.0	–	7.5	–	1.8	–	*0.0010
Number of Teachers (mean, *SD*)	4.0	4.7	4.2	4.3	1.4	0.8	<0.0001*
Percent of Teachers with Bachelor’s degree or higher (mean%, *SD*)	20.8	33.3	9.4	21.4	11.2	29.5	<0.0001*
Number of Additional Supporting Staff (mean, *SD*)	4.6	6.8	2.2	2.3	0.6	0.8	<0.0001*
Number of Total Classrooms (mean, *SD*)	4.5	4.9	6.1	3.4	1.4	1.3	<0.0001*
Number of 3-to-5-Year-Old Classrooms (mean, *SD*)	3.4	4.6	2.2	1.1	1.3	1.4	<0.0001*
Number of Total Children (mean, *SD*)	61.5	79.6	66.5	45.2	8.9	4.1	<0.0001*
Number of 3-to-5-Year-Old Children (mean, *SD*)	52.9	80.4	25.9	19.0	3.8	2.5	<0.0001*

Percent of 3-to-5-Year-Old Child Race/Ethnicity (mean%, *SD*)							
Hispanic	16.4	18.0	5.2	6.8	4.3	13.6	<0.0001*
American Indian	16.7	21.4	12.5	17.2	13.7	25.1	0.0019*
Asian	1.7	7.3	1.5	4.2	0.8	4.9	<0.0001*
Black or African American	10.5	13.8	10.9	18.3	10.7	24.4	<0.0001*
Native Hawaiian or Pacific Islander	1.1	6.5	0.5	1.9	1.4	10.1	0.0402*
White or Caucasian	44.9	26.9	54.6	30.6	57.0	38.7	0.0191*
Mixed race	11.1	11.6	9.6	15.7	9.2	20.3	<0.0001*
Other	0.0	0.0	0.4	2.4	0.7	8.0	0.1234
Non-specified	0.5	3.6	7.6	22.2	5.1	18.2	0.0090*
Serve Children on SNAP (%)	87.0	–	28.0	–	68.8	–	<0.0001*
Serve Children on WIC (%)	94.4	–	32.3	–	59.5	–	<0.0001*
Serve Children Struggling with Hunger (%)	53.7	–	69.2	–	97.1	–	<0.0001*
Serve Children Lacking Access to Healthy Foods at Home (%)	61.1	–	56.7	–	90.7	–	<0.0001*
NAEYC Accredited (%)	28.3	–	11.4	–	6.2	–	<0.0001*

Professional Program Participation (%)							
CACFP	98.5	–	62.2	–	88.7	–	<0.0001*
Go NAPSACC	7.4	–	3.7	–	1.8	–	0.1325
Healthy Body, Healthy Minds	5.5	–	4.4	–	3.1	–	0.5477
Happy Healthy Homes	3.7	–	0.0	–	6.2	–	0.0020*
Certified Early Childhood	20.3	–	11.9	–	6.2	–	0.0120*
Food Preparation Occurs On-Site (%)	81.4	–	88.0	–	97.4	–	0.0009*

Methods for Purchasing Center Foods (%)							
In-person shopping at a store	13.2	–	33.9	–	77.5	–	<0.0001*
Online ordered then picked up in person	1.8	–	24.3	–	16.8	–	<0.0001*
Online and delivered	43.4	–	28.2	–	5.0	–	<0.0001*
Over the phone with a vendor	41.5	–	13.4	–	0.6	–	<0.0001*
Roundtrip Miles to Purchasing Center Foods (mean, *SD*)	11.6	13.7	15.5	21.3	18.7	22.6	0.1018

Person Responsible for Center Meal Planning (%)							
Owner/Director	9.2	–	40.8	–	95.0	–	<0.0001*
Cook or Chef	33.3	–	50.9	–	3.1	–	<0.0001*
Catering Company	3.7	–	0.6	–	0.0	–	<0.0001*
Dietician	27.7	–	2.5	–	0.0	–	<0.0001*
Parents/Guardians	0.0	–	1.2	–	0.0	–	<0.0001*
^€^Other	25.9	–	3.7	–	1.8	–	<0.0001*
Located within a “low proximity” area (%)	24.0	–	27.6	–	36.8	–	0.1066
Distance in Miles to Nearest Grocery Store (mean%, *SD*)	2.2	3.3	1.5	2.6	2.3	3.1	0.0138*

Percent Urban/Rural within Census Tract (%)							
Urban	44.4	–	61.6	–	61.2	–	0.0667
Rural	55.5	–	38.3	–	38.7	–	0.0667

*indicates significant difference among groups (*p*-value < 0.05). CBC = Community-Based Childcare; FCCH = Family Child Care Homes; *Center Hours survey item allowed for multiple response; CACFP = Child and Adult Care Food Program (CACFP) by USDA; Go NAPSACC = Nutrition and Physical Activity Self-Assessment for Child Care. ^€^”Other” responses were written in by participants, and included but were not limited to persons such as: nutrition manager, public school, assistant director, and self.

ECEs were primary full-time; several Head Start centers reported having both full-time and part-time programs operating in a single facility (Table 1). Compared with CBCs and FCCHs, Head Start centers reported the shortest roundtrip distance in miles traveled to purchase foods, and highest frequency of a dietitian being responsible for planning meals served to young children. Compared with Head Starts and FCCHs, CBCs demonstrated the highest frequency of having a cook or chef responsible for center meal planning. Compared with Head Starts and CBCs, FCCHs reported the highest frequency of purchasing foods in-person at a store, with the owner/director mostly being responsible for program meal planning; and reported the longest geographic distance in miles to the nearest grocery store. Although reported roundtrip distance traveled to purchase foods and likelihood of being in a low proximity area varied between ECE contexts, results were not statistically significant.

### 3.1 ECE context and classroom nutrition practices

Across the three ECE contexts, classroom nutrition practices and reported barriers to implementation varied significantly (Table 2). Across all ECE contexts, average sub-section scores for classroom nutrition practices indicated that Oklahoma ECEs were mostly meeting minimum recommended standards (average score of at least two for each item). Head Starts reported the highest implementation of overall nutrition practices, with notably highest scores specifically for Supporting Healthy Eating, Nutrition Education, and Nutrition Policy sub-sections compared with CBCs and FCCHs. On the other hand, FCCHs demonstrated lowest NAPSACC Nutrition Total Score and multiple sub-section scores. However, FCCHs did report healthiest practices for the Meats, Fats and Grains sub-section.

**Table 2:** Classroom nutrition practice scores and barriers among Oklahoma ECE programs participating in the Communities and Classroom Health Survey in 2019–2020, by child care context.

	**Head Start** **(*n* = 54)**		**CBC** **(*n* = 159)**		**FCCH** **(*n* = 160)**		
Classroom Nutrition Practice Scores (mean, *SD*)							
	mean	*SD*	mean	*SD*	mean	*SD*	*p*-value
*NAPSACC Nutrition Total Score	29.2	2.7	26.4	3.4	25.5	3.4	<0.0001*
1. Fruits and Vegetables Served	3.1	0.5	3.1	0.5	3.1	0.5	0.3606
2. Meats, Fats and Grains	3.0	0.4	3.0	0.4	3.2	0.4	0.0002*
3. Beverages Served	3.3	0.3	3.3	0.3	3.4	0.3	0.3210
4. Menus and Variety	2.7	0.7	2.7	0.6	2.6	0.6	0.3476
5. Feeding Practices	3.3	0.5	3.3	0.5	3.3	0.5	0.3212
6. Foods Offered Outside of Regular Meals	2.7	0.9	2.6	0.7	2.2	0.7	<0.0001*
7. Supporting Healthy Eating	3.6	0.3	2.9	0.5	2.7	0.5	<0.0001*
8. Nutrition Education	3.4	0.5	2.2	0.7	2.1	0.8	<0.0001*
9. Nutrition Policy	3.7	0.7	2.9	1.1	2.6	1.3	<0.0001*

Barriers to Serving Healthier Meals and Snacks (%yes)							
	%	–	%	–	%	–	*p*-value
Not enough money for purchasing healthier meals	16.6	–	37.7	–	28.1	–	0.0100*
Lack of control over the types of meals served/delivered	31.4	–	14.7	–	2.5	–	<0.0001*
Lack of knowledge on preparing healthier foods	9.2	–	12.5	–	4.3	–	0.0321*
Lack of time to prepare healthier foods	20.3	–	20.1	–	15.0	–	0.4338
Children would not like the taste of healthier meals	11.1	–	36.7	–	35.0	–	0.0015*
Concern for food waste due to child preference	14.8	–	31.6	–	40.5	–	0.0022*
Parents do not want children to be served healthy foods	0.0	–	4.4	–	4.4	–	0.2896
Limited space for food storage	22.2	–	22.6	–	22.0	–	0.9908
Lack of availability of healthy foods in my area	14.8	–	12.6	–	8.8	–	0.3790
Lack of support from other providers	9.2	–	6.9	–	2.5	–	0.0852
Other areas in our program have higher priority	9.2	–	10.2	–	10.0	–	0.9779
So many recommendations to know which to follow	3.7	–	19.8	–	13.8	–	0.0155*
Unsure which foods can be reimbursed through CACFP	5.5	–	18.0	–	10.6	–	0.0319*
Weekly schedule limits time to shop	15.0	–	26.1	–	25.9	–	0.2293

Barriers to Healthful Mealtime Practices (%yes)							
	%	–	%	–	%	–	*p*-value
Providers do not have time to sit with children at meals	7.4	–	17.0	–	35.6	–	<0.0001*
Not enough providers to sit with children during meals	9.2	–	10.1	–	24.5	–	0.0008*
Not enough money to purchase meals for providers	7.4	–	22.0	–	13.1	–	0.0167*
Providers unsure how to support children’s healthy eating	9.2	–	16.9	–	10.6	–	0.1657
Providers do not like the taste of the healthy foods	20.3	–	14.4	–	9.3	–	0.0951
Providers have dietary restrictions	16.6	–	16.3	–	13.1	–	0.6750
Providers are uncertain how to handle picky eaters	12.9	–	16.3	–	17.5	–	0.7380
Mealtimes with children are stressful/chaotic	20.3	–	22.7	–	22.6	–	0.9293
If children serve themselves, they would not eat/drink enough	14.8	–	15.0	–	25.9	–	0.0325*
If children serve themselves, they would eat/drink too much	16.6	–	13.2	–	24.5	–	0.0339*
Too much of a mess if children serve themselves	19.2	–	30.1	–	40.6	–	0.0109*

*indicates significant difference among groups (*p*-value < 0.05). NAPSACC = Nutrition and Physical Activity Self-Assessment for Child Care. *Higher NAPSACC scores indicate higher frequency or healthier degree of implementing nutrition requirements and best practices.

For reported barriers to serving healthier meals and snacks, Head Starts were most likely to report perceived lack of control over the types of meals served, and least likely to report most other barriers compared with CBCs and FCCHs. Compared with Head Starts and FCCHs, CBCs were most likely to report barriers such as not having enough money to cover the cost of purchasing foods, lack of knowledge on preparing healthier foods, not knowing what recommendations to follow, and being unsure of which foods are reimbursed by CACFP. Compared with Head Starts and CBCs, FCCHs were most likely to report barriers including concern for food waste due to children not liking the taste of healthier foods.

For reported barriers to implementing healthful mealtime practices, Head Starts consistently reported perceived barriers less frequently compared to CBCs and FCCHs. Compared with Head Starts and FCCHs, CBCs were most likely to report barriers, including not having enough money to cover costs of serving foods to providers and providers being unsure of how to encourage children’s healthy eating. Compared with Head Starts and CBCs, FCCHs were most likely to report barriers, including providers not having enough time to sit with children during meals, not enough providers to sit with children during meals, and concern that if children served themselves, they would not eat/drink enough, would eat/drink too much, and would make too much of a mess.

### 3.2 Proximity to grocery stores, classroom nutrition practices, and barriers

Location of the ECE within a low proximity area was not related to differing classroom nutrition practice scores across all ECE contexts (Table 3). There was no difference in frequency of reported barriers based on proximity to grocery stores for Head Starts and CBCs. However, FCCHs located within a low proximity area were more likely to report perceived concern for food waste (55.1 %), compared with FCCHs located within accessible proximity of grocery store (32.0 %; *p* < 0.004).

**Table 3:** Differences in classroom nutrition practice scores and barriers based on proximity to grocery stores among Oklahoma ECE programs participating in the Communities and Classroom Health Survey in 2019–2020, by child care context.

	**Head Start** **(*n* = 54)**				**CBC** **(*n* = 159)**				**FCCH** **(*n* = 160)**			
	Low Proximity (*n* = 13)		Access to Grocery (*n* = 41)		Low Proximity (*n* = 44)		Access to Grocery (*n* = 159)		Low Proximity (*n* = 59)		Access to Grocery (*n* = 101)	
Classroom Nutrition Practice Scores (mean, *SD*)												
	mean	*SD*	mean	*SD*	mean	*SD*	mean	*SD*	mean	*SD*	mean	*SD*
*NAPSACC Nutrition Total Score	29.4	2.2	29.2	2.8	26.6	3.2	26.3	3.4	25.5	3.3	25.6	3.5
1. Fruits and Vegetables Served	3.0	0.5	3.1	0.4	3.1	0.4	3.0	0.5	3.2	0.5	3.1	0.4
2. Meats, Fats and Grains	3.1	0.4	3.0	0.4	3.1	0.3	3.0	0.4	3.3	0.4	3.2	0.4
3. Beverages Served	3.3	0.3	3.3	0.3	3.3	0.3	3.3	0.3	3.4	0.3	3.3	0.3
4. Menus and Variety	2.9	0.5	2.7	0.7	2.7	0.6	2.7	0.7	2.6	0.6	2.6	0.6
5. Feeding Practices	3.3	0.3	3.3	0.5	3.4	0.4	3.3	0.5	3.3	0.5	3.3	0.6
6. Foods Offered Outside of Regular Meals	2.8	0.8	2.7	0.9	2.7	0.8	2.5	0.7	2.1	0.8	2.3	0.7
7. Supporting Healthy Eating	3.6	0.4	3.6	0.3	2.9	0.5	2.9	0.5	2.7	0.6	2.7	0.5
8. Nutrition Education	3.3	0.6	3.4	0.4	2.1	0.8	2.2	0.7	2.0	0.8	2.1	0.8
9. Nutrition Policy	3.8	0.5	3.6	0.8	2.8	1.2	3.0	1.1	2.5	1.3	2.6	1.3

Barriers to Serving Healthier Meals and Snacks (%yes)												
	%	–	%	–	%	–	%	–	%	–	%	–
Not enough money to cover the cost of purchasing healthier meals	7.6	–	19.5	–	31.8	–	40.0	–	30.5	–	26.7	–
Lack of control over the types of meals served/delivered	38.4	–	29.2	–	11.9	–	15.7	–	3.3	–	1.9	–
Lack of knowledge on preparing healthier foods and beverages	0.0	–	12.2	–	13.6	–	12.1	–	5.0	–	3.9	–
Lack of time to prepare healthier foods and beverages	7.6	–	24.3	–	25.0	–	18.2	–	20.3	–	11.8	–
Children would not like the taste of healthier meals and beverages	7.6	–	12.2	–	36.3	–	34.5	–	42.3	–	33.3	–
Concern for food waste due to children not eating healthier meals	15.3	–	14.6	–	27.2	–	33.3	–	**55.1**	–	32.0*	–
Parents do not want children to be served healthier foods	0.0	–	0.0	–	0.0	–	6.1	–	6.7	–	3.0	–
Limited space for food storage (i.e., refrigerator and cabinet space)	23.0	–	21.9	–	18.1	–	24.3	–	30.5	–	17.0	–
Lack of availability of healthy foods in my area	15.3	–	14.6	–	6.9	–	14.7	–	11.8	–	7.0	–
Lack of support from other providers	7.6	–	9.7	–	6.8	–	7.0	–	5.0	–	1.0	–
Other areas in our program have higher priority than nutrition	0.0	–	12.2	–	4.5	–	12.5	–	13.5	–	8.0	–
So many recommendations providers do not know which to follow	0.0	–	3.7	–	20.9	–	19.4	–	22.0	–	9.0	–
Unsure which foods can be reimbursed through CACFP	0.0	–	7.3	–	21.4	–	16.8	–	8.4	–	12.0	–
Weekly schedule limits time to shop more than once per week	23.0	–	12.5	–	20.0	–	28.4	–	28.5	–	24.4	–

Barriers to Healthful Mealtime Practices (%yes)												
	%	–	%	–	%	–	%	–	%	–	%	–
Providers do not have time to sit with children during meals	7.6	–	7.3	–	18.1	–	16.6	–	38.9	–	33.6	–
Not enough providers to sit with children during meals	0.0	–	9.2	–	11.3	–	9.6	–	23.7	–	25.0	–
Not enough money to cover the cost of serving meals to providers	7.6	–	7.3	–	22.7	–	21.7	–	13.5	–	12.8	–
Providers are unsure how to encourage children’s healthy eating	0.0	–	12.2	–	13.6	–	18.2	–	12.0	–	9.9	–
Providers do not like the taste of the healthy foods	15.3	–	21.9	–	9.0	–	16.5	–	11.8	–	7.9	–
Providers have dietary restrictions	15.3	–	17.0	–	15.9	–	16.5	–	15.2	–	11.8	–
Providers are uncertain how to handle children who refuse foods	15.3	–	12.2	–	11.3	–	18.2	–	22.0	–	14.8	–
Providers feel mealtimes with children are stressful/chaotic	23.0	–	19.5	–	15.9	–	25.4	–	25.4	–	21.0	–
If children serve themselves, they would not eat/drink enough	15.3	–	14.6	–	15.9	–	14.7	–	29.3	–	24.0	–
If children serve themselves, they would eat/drink too much	23.0	–	14.6	–	6.8	–	15.7	–	18.6	–	28.0	–
If children serve themselves, they will make too much of a mess	23.0	–	17.9	–	27.2	–	31.2	–	46.4	–	37.2	–

*p-value < 0.004; indicates significant association after Benjamini Hochberg correction for False Discovery Rate. ECE = center for Early Childhood Education; CBC = Community-Based Childcare; FCCH = Family Child Care Homes; NAPSACC = Nutrition and Physical Activity Self-Assessment for Child Care. *Higher NAPSACC scores indicate higher frequency or healthier degree of implementing nutrition requirements and best practices.

Differences in classroom nutrition practices by proximity to grocery stores were further stratified by CACFP participation in CBCs and FCCHs (Table 4). We hypothesized that ECEs that did not participate in the CACFP might be more vulnerable to the influence of the surrounding community environment. However, similar to non-stratified study findings, classroom nutrition practice scores did not vary among those located in a low proximity area versus those that were not, regardless of CACFP participation. Interestingly, among FCCHs participating in the CACFP only, those located within a low proximity area were more likely to report perceived concern for food waste (57.6 %) than did FCCHs located within accessible proximity of grocery store (31.8 %; *p* < 0.004).

**Table 4:** Differences in classroom nutrition practice scores and barriers based on proximity to grocery stores among Oklahoma CBCs (*n* = 159) and FCCHs (*n* = 162) in 2019–2020, stratified by participation in the Child and Adult Care Food Program (CACFP).

	CBCs (*n* = 159)								FCCHS (*n* = 160)							
	CACFP (*n* = 99; 62.2 %)				Non-CACFP (*n* = 60; 37.7 %)				CACFP (*n* = 142; 88.7 %)				Non-CACFP (*n* = 18; 11.2 %)			
	Low Proximity (*n* = 32)		Access to Grocery (*n* = 67)		Low Proximity (*n* = 12)		Access to Grocery (*n* = 48)		Low Proximity (*n* = 53)		Access to Grocery (*n* = 89)		Low Proximity (*n* = 6)		Access to Grocery (*n* = 12)	
Classroom Nutrition Practice Scores (mean, *SD*)																
	mean	*SD*	mean	*SD*	mean	*SD*	mean	*SD*	mean	*SD*	mean	*SD*	mean	*SD*	mean	*SD*
*NAPSACC Nutrition Total Score	26.8	2.9	26.9	3.3	26.0	3.8	25.5	3.5	25.7	3.3	25.6	3.3	23.7	2.8	25.1	4.8
1. Fruits and Vegetables Served	3.2	0.4	3.1	0.4	3.0	0.5	2.9	0.5	3.2	0.5	3.1	0.4	3.2	0.3	3.1	0.5
2. Meats, Fats, and Grains	3.2	0.3	3.1	0.3	2.9	0.4	2.9	0.6	3.3	0.4	3.2	0.4	3.0	0.3	3.0	0.5
3. Beverages Served	3.3	0.2	3.3	0.3	3.4	0.3	3.2	0.3	3.4	0.3	3.3	0.3	3.2	0.2	3.3	0.3
4. Menus and Variety	2.7	0.5	2.7	0.6	2.9	0.6	2.6	0.8	2.6	0.6	2.6	0.6	2.5	0.8	2.8	0.8
5. Feeding Practices	3.3	0.4	3.4	0.5	3.4	0.5	3.3	0.6	3.3	0.5	3.3	0.6	3.3	0.4	3.2	0.5
6. Foods Offered Outside of Regular Meals	2.7	0.8	2.5	0.7	2.7	0.5	2.6	0.7	2.1	0.8	2.2	0.7	1.9	0.8	2.4	0.8
7. Supporting Healthy Eating	2.9	0.5	3.0	0.6	3.0	0.5	2.8	0.5	2.7	0.6	2.7	0.5	2.8	0.2	2.6	0.5
8. Nutrition Education	2.1	0.8	2.3	0.8	2.1	0.9	2.0	0.6	2.1	0.8	2.1	0.8	1.6	0.6	1.8	0.9
9. Nutrition Policy	3.0	1.1	3.1	1.0	2.4	1.4	2.8	1.2	2.6	1.3	2.6	1.3	2.0	1.2	2.4	1.3

Barriers to Serving Healthier Meals and Snacks (%yes)																
	%	–	%	–	%	–	%	–	%	–	%	–	%	–	%	–
Not enough money to cover the cost of purchasing healthier meals	34.3	–	37.3	–	25.0	–	43.7	–	33.9	–	25.8	–	0.0	–	33.3	–
Lack of control over the types of meals served/delivered	9.6	–	13.4	–	18.1	–	19.1	–	3.7	–	2.2	–	0.0	–	0.0	–
Lack of knowledge on preparing healthier foods and beverages	15.6	–	11.9	–	8.3	–	12.5	–	5.6	–	4.4	–	0.0	–	0.0	–
Lack of time to prepare healthier foods and beverages	18.7	–	19.4	–	41.6	–	16.6	–	20.7	–	8.9	–	16.6	–	33.3	–
Children would not like the taste of healthier meals and beverages	37.5	–	36.3	–	33.3	–	31.9	–	43.4	–	31.0	–	33.3	–	50.0	–
Concern for food waste due to children not eating healthier meals	18.7	–	36.3	–	50.0	–	29.1	–	**57.6**	–	31.8*	–	33.3	–	33.3	–
Parents do not want children to be served healthier foods	0.0	–	5.9	–	0.0	–	6.3	–	7.5	–	3.4	–	0.0	–	0.0	–
Limited space for food storage (i.e. refrigerator and cabinet space)	12.5	–	29.8	–	33.3	–	16.6	–	33.9	–	15.9	–	0.0	–	25.0	–
Lack of availability of healthy foods in my area	9.3	–	17.9	–	0.0	–	10.4	–	13.2	–	6.8	–	0.0	–	8.3	–
Lack of support from other providers	9.3	–	9.0	–	0.0	–	3.3	–	5.6	–	1.1	–	0.0	–	0.0	–
Other areas in our program have higher priority than nutrition	6.2	–	13.6	–	0.0	–	8.6	–	15.0	–	6.8	–	0.0	–	16.6	–
So many recommendations providers do not know which to follow	19.3	–	24.6	–	25.0	–	12.5	–	22.6	–	10.2	–	16.6	–	0.0	–
Unsure which foods can be reimbursed through CACFP	16.1	–	18.1	–	36.3	–	14.8	–	9.4	–	11.3	–	0.0	–	16.6	–
Weekly schedule limits time to shop more than once per week	14.2	–	41.2	–	33.3	–	10.8	–	32.0)	–	24.4)	–	0.0	–	25.0	–

Barriers to Healthful Mealtime Practices (%yes)																
	%	–	%	–	%	–	%	–	%	–	%	–	%	–	%	–
Providers do not have time to sit with children during meals	15.6	–	10.6	–	25.0	–	25.0	–	41.5	–	31.4	–	16.6	–	50.0	–
Not enough providers to sit with children during meals	9.3	–	7.4	–	16.6	–	12.7	–	24.5	–	23.8	–	16.6	–	33.3	–
Not enough money to cover the cost of serving meals to providers	18.7	–	22.3	–	33.3	–	20.8	–	15.0	–	14.6	–	0.0	–	0.0	–
Providers are unsure how to encourage children’s healthy eating	12.5	–	19.4	–	16.6	–	16.6	–	11.5	–	11.2	–	5.5	–	0.0	–
Providers do not like the taste of the healthy foods	9.3	–	19.4	–	8.3	–	12.5	–	13.2	–	8.9	–	0.0	–	0.0	–
Providers have dietary restrictions	12.5	–	14.9	–	25.0	–	18.7	–	16.9	–	11.2	–	0.0	–	16.6	–
Providers are uncertain how to handle children who refuse foods	9.3	–	16.4	–	16.6	–	20.8	–	20.7	–	15.7	–	33.3	–	8.3	–
Providers feel mealtimes with children are stressful/chaotic	15.6	–	24.2	–	16.6	–	27.0	–	8.5	–	20.4	–	50.0	–	50.0	–
If children serve themselves, they would not eat/drink enough	15.6	–	16.4	–	16.6	–	12.5	–	30.7	–	27.2	–	16.6	–	0.0	–
If children serve themselves, they would eat/drink too much	6.2	–	16.6	–	1.6	–	14.5	–	20.7	–	29.2	–	0.0	–	18.1	–
If children serve themselves, they will make too much of a mess	28.1	–	35.9	–	25.0	–	25.0	–	46.0	–	36.5	–	50.0	–	41.6	–

*p-value < 0.004; indicates significant association after Benjamini Hochberg correction for False Discovery Rate. CBC = Community-Based Childcare; FCCH = Family Child Care Homes; NAPSACC = Nutrition and Physical Activity Self-Assessment for Child Care. *Higher NAPSACC scores indicate higher frequency or healthier degree of implementing nutrition requirements and best practices.

## 4 Discussion

The present study aimed to determine how community food environment, i.e., proximity to grocery stores, was associated with ECE classroom nutrition practices and barriers by ECE context (Head Starts, CBCs, and FCCHs). Proximity to grocery stores was not related to classroom nutrition practices within any ECE context; findings were consistent with those analyzing relationships between physical activity built environments with activity-promoting classroom practices for the same sample (Williams et al., 2021). Results emphasize the importance of ECEs to serve as a protective resource for young children vulnerable to health risk. FCCHs located within low proximity areas did report higher frequency of barriers to implementing those practices compared with FCCHs within proximity of a nearby grocery store, specifically concern for food waste. To our knowledge, this is the first study to report on how proximity to grocery stores of ECEs impacts classroom health practices and experiences of staff across various ECE contexts. Finally, consistent with similar studies in observing that Head Start centers report healthiest frequency/degree of classroom nutrition practices (Dev et al., 2013, Whitaker et al., 2009), with FCCHs demonstrating the lowest (Dev et al., 2020, Benjamin-Neelon et al., 2018).

The present findings provide insight into how logistical and organizational factors related to serving of healthful foods vary by ECE context. Specifically, Head Start centers travel the shortest reported distance to purchase food, and more often have a dietitian employed to plan center meals and are least likely to be located within a low proximity area. Conversely, FCCHs are most likely to purchase foods in-person at a store versus online or over the phone with a vendor, with the owner/director being primarily responsible for center meal planning and travel the furthest distance to purchase foods. Previous research in CBCs and FCCHs confirmed that FCCHs are more likely to purchase foods in person, and that ECEs that purchase foods online serve meals of higher nutritional quality than do those using in-person procurement methods (Lazarus et al., 2018). While a report published by the National Food Service Management Institute recommendations that CACFP-participating centers purchase foods with a vendor, and that positive vendor relationships are influential for ensuring consistent quality of foods delivered and purchasing flexibility (Perkins, 2004), this option may not be cost-efficient or plausible for FCCHs and small CBCs. Previous qualitative work also suggests that overall, providers feel that fresh foods are of lower quality when purchased with vendors than when shopping in person at a grocery store (Lynch and Batal, 2011). Given that online purchasing options may support implementation of healthier classroom nutrition practices (Lazarus et al., 2018), FCCHs and other ECEs with barriers to utilizing such procurement methods may benefit from training and technical assistance on how to efficiently work with food vendors and/or how to nutritionally maximize in-person shopping.

Classroom nutrition practices did not differ by proximity to grocery stores, regardless of CACFP participation. The results were contrary to our hypothesis, which was primarily informed by residential studies, those in elementary-, middle-, and high-school students (Drewnowski and Poverty, 2004, Cutumisu et al., 2017, Williams et al., 2014). Limited work conducted in ECE settings have suggested a positive relationship between proximity to food outlets and quality of foods served to children in English pre-schools and U.S. family child care homes (Burgoine and Gallis, 2017, Braun et al., 2022). Qualitative studies in ECEs have identified predictors of serving healthful foods to young children, which were primarily individual/provider-level characteristics (knowledge and self-efficacy) or at the larger organization-level (support among the local ECE community and resources for nutritional information) (Lynch and Batal, 2011). Previous studies have described influence of policy and standards on classroom health, with more desirable classroom nutrition practices reported among ECEs with more stringent licensing requirements, Head Start performance standards, and CACFP participation which varies greatly across CBCs and FCCHs (Dev et al., 2013, Zaltz et al., 2020, Erinosho et al., 2018). Thus, the present study findings add to previous literature to support that ECEs, regardless of CACFP participation, may serve as a protective micro-environment to support health for children and families who are more vulnerable to the health environments of their nearby residing communities. This finding underscores the importance of understanding how best to support implementation of health practices for ECE through within-center and policy-focused intervention.

In CACFP-participating FCCHs only, nearly twice as many directors from FCCHs in low proximity areas reported concern for food waste as a barrier for serving healthful foods, compared to those within accessible proximity of grocery store. Current and previous findings demonstrate that commonly reported barriers to nutrition practice implementation among FCCH providers are typically those related to lack of resources (Lindsay et al., 2015, Zaltz et al., 2018, Tovar et al., 2015). Further, FCCHs are located within residential areas and are more likely to purchase center foods in-person at a grocery store. Consistent with previous literature, present study findings indicate overall differing barriers to nutrition practice implementation reported across ECE contexts (Lindsay et al., 2015, Zaltz et al., 2018, Tovar et al., 2015). In the present sample, FCCHs reported highest frequency of concern for food waste due to child preference, lack of time and staff, and concern for the additional mess related to serving meals family style as barriers to implementing mealtime best practices. Such concerns experienced by FCCHs could be attributed to the additional budgetary, time, scheduling, and organizational constraints incurred from FCCH providers’ multiple responsibilities as ECE director, owner, teacher, and food staff (Lindsay et al., 2015, Dev et al., 2017). These findings add to previous work by suggesting that experiences and perceptions of FCCH providers may be more susceptible to influence of the community food environment, especially when implementing nutrition practices required by CACFP standards. Tailored resources through CACFP or agencies providing ECE support and technical assistance to support FCCH providers in low-access communities should be considered to alleviate common barriers to implementing nutrition practices. While nutrition practices did not differ based on proximity to grocery stores, addressing community-specific barriers may be a useful strategy to improve implementation for mealtime best practices with low implementation in FCCHs overall.

The strengths and limitations of the present study warrant discussion. Strengths of the study included use of grocery store data that were validated in person and use of a representative statewide sample including each of the three primary ECE contexts. Limitations include that due to the cross-sectional study design, causality cannot be inferred. There is a temporal difference in data collection, specifically that locations of grocery stores were determined by in-person audit that was conducted in 2016 and therefore may not fully represent locations of grocery stores surrounding ECEs at the time the statewide survey was distributed in 2019–2020. Methods for ECEs purchasing center foods in-person differed significantly by proximity to grocery stores and ECE context; however, being outside the scope of present study aims, these differences were not accounted for statistically. Data were self-reported by ECE directors and could be subject to social desirability, particularly among those participating in CACFP with assumed knowledge of standards and best practices. Further, GIS-determined proximity to grocery stores may not fully reflect actual shopping route and habits of ECEs. The current study sample may additionally be subject to selection bias, where those who participated are those implementing healthier practices. Cell size was relatively limited in stratified analyses, and therefore should be interpreted with caution. Finally, the primary respondent was the ECE director, who may not have complete knowledge of current classroom activities or provider barriers; this limitation may be more pertinent in Head Starts and CBCs than in FCCHs, where directors typically serve in all roles simultaneously. However, respondents were instructed to defer to the staff with most accurate insight on that practice. Primary study strengths were use of a statewide sample, validated data on grocery store locations, and the NAPSACC tool to assess classroom nutrition practices is widely used and has been validated against nutrition practices observed multiple days in-classroom by trained research personnel (Benjamin et al., 2007).

## 5 Conclusion

Present study findings do not demonstrate differences in classroom nutrition practices, including foods served and mealtime best practices, based on proximity to grocery stores as a marker of the community food environment. However, for FCCHs only, location within a “low proximity” area was associated with reporting higher frequency of barriers to serving healthful food and beverages. This relationship remained significant among those FCCHs participating in the CACFP. Findings on implementation of classroom nutrition practices indicate that ECEs may be protective overall of the surrounding community environment, unlike schools or homes (Drewnowski and Poverty, 2004, Cutumisu et al., 2017, Williams et al., 2014), though community-specific strategies to reduce prominent barriers for implementing nutrition practices should be considered. Differences in how ECEs may interact with their surrounding community environment may be attributable to differences in allotted resources for implementing health practices, as well as food preparation methods, meal planning, and food purchasing. Future studies should seek to examine whether food prep/planning methods influence implementation of nutrition practices, and whether perceived constructs of community food access, including reported distance to purchase foods and access-related barriers, are related to classroom nutrition practices and barriers for differing contexts of ECEs. Finally, our findings suggest a need for future research and policy implementation, potentially through CACFP, to understand how to provide support for FCCHs residing in low-access areas having trouble implementing classroom health practices.

## CRediT authorship contribution statement

**Bethany D. Williams:** Conceptualization, Methodology, Data curation, Funding acquisition, Investigation, Software, Formal analysis, Project administration, Visualization, Writing – original draft, Writing – review & editing. **Susan B. Sisson:** Conceptualization, Methodology, Data curation, Funding acquisition, Investigation, Software, Supervision, Writing – review & editing. **Bryce C. Lowery:** Conceptualization, Methodology, Supervision, Writing – review & editing. **Dipti A. Dev:** Conceptualization, Methodology, Supervision, Writing – review & editing. **Diane M. Horm:** Conceptualization, Methodology, Supervision, Writing – review & editing. **Janis E. Campbell:** Conceptualization, Methodology, Supervision, Writing – review & editing. **Denise A. Finneran:** Conceptualization, Methodology, Supervision, Writing – review & editing. **Jennifer Graef-Downard:** Conceptualization, Methodology, Supervision, Writing – review & editing.

## Declaration of Competing Interest

The authors declare that they have no known competing financial interests or personal relationships that could have appeared to influence the work reported in this paper.

## References

[b0005] Andreyeva T., Henderson K.E. (2018). Center-reported adherence to nutrition standards of the child and adult care food program. Child Obes..

[b0010] Bassett R., Chapman G.E., Beagan B.L. (2008). Autonomy and control: the co-construction of adolescent food choice. Appetite.

[b0015] Benjamin S.E., Neelon B., Ball S.C., Bangdiwala S.I., Ammerman A.S., Ward D.S. (2007). Reliability and validity of a nutrition and physical activity environmental self-assessment for child care. Int. J. Behav. Nutr. Phys. Activity.

[b0020] Benjamin Neelon S.E., Briley M.E. (2011). Position of the American Dietetic Association: benchmarks for nutrition in child care. J. Am. Dietetic Assoc..

[b0025] Benjamin-Neelon S.E., Vaughn A.E., Tovar A., Ostbye T., Mazzucca S., Ward D.S. (2018). The family child care home environment and children’s diet quality. Appetite.

[b0030] Birch L. (1998). Development of food acceptance patterns in the first years of life. Proc. Nutr. Soc..

[b0035] Birch L.L., Fisher J.O. (1998). Development of eating behaviors among children and adolescents. Pediatrics.

[b0040] Braun L.M., Ward D., Hales D., Vaughn A., Erinosho T. (2022). Food outlet density, distance, and food quality offered to preschool-aged children at family child care homes. J. Nutr. Educ. Behav..

[b0045] Burgoine T., Gallis J.A.T.L.P., Monsivais P., Benjamin Neelon S.E. (2017). Association between distance to nearest supermarket and provision of fruits and vegetables in English nurseries. Health Place.

[b0050] Centers for Disease Control and Prevention. Census Tract Level State Maps of the Modified Retail Food Environment Index (mRFEI) 2018 [cited 2019 January 5]. Available from: ftp://ftp.cdc.gov/pub/Publications/dnpao/census-tract-level-state-maps-mrfei_TAG508.pdf.

[b0055] Cutumisu N., Traore I., Paquette M.C., Cazale L., Camirand H., Lalonde B. (2017). Association between junk food consumption and fast-food outlet access near school among Quebec secondary-school children: findings from the Quebec Health Survey of High School Students (QHSHSS) 2010-11. Public Health Nutr..

[b0060] Dev A., Dipti M., McBride B.A., Team T.S.K.R. (2013). Academy of nutrition and dietetics benchmarks for nutrition in child Care 2011: are child-care providers across contexts meeting recommendations?. Acad. Nutr. Dietetics.

[b0065] Dev DA, Carraway-Stage V, Schober DJ, McBride BA, Kok CM, Ramsay S. Implementing the academy of nutrition and dietetics benchmarks for nutrition education for children: child-care providers’ perspectives. J. Acad. Nutr. Dietetics. 2017;117(12):1963-71 e2. Epub 2017/08/29. doi: 10.1016/j.jand.2017.07.001. PubMed PMID: 28844891. 10.1016/j.jand.2017.07.001 28844891

[b0070] Dev D.A., Garcia A.S., Dzewaltowski D.A., Sisson S., Franzen-Castle L., Rida Z. (2020). Provider reported implementation of nutrition-related practices in childcare centers and family childcare homes in rural and urban Nebraska. Prev. Med. Rep..

[b0075] Dev D.A., Speirs K.E., McBride B.A., Donovan S.M., Chapman-Novakofski K. (2014). Head Start and child care providers’ motivators, barriers and facilitators to practicing family-style meal service. Early Childhood Res. Quart..

[b0080] Drewnowski A., Specter S.E. (2004). Poverty and obesity: the role of energy density and energy costs. Am. J. Clin. Nutr..

[b0085] Erinosho T., Vaughn A., Hales D., Mazzucca S., Gizlice Z., Ward D. (2018). Participation in the child and adult care food program is associated with healthier nutrition environments at family child care homes in Mississippi. J. Nutr. Educ. Behav..

[b0090] Frisvold D.E., Lumeng J.C. (2011). Expanding exposure can increasing the daily duration of head start reduce childhood obesity?. J. Hum. Resourc..

[b0095] Garcia A.S., Dev D.A., Stage V.C. (2018). Predictors of parent engagement based on child care providers’ perspectives. J. Nutr. Educ. Behav..

[b0100] Dietary Guidelines for Americans 2015-2020. 2015.

[b0105] Gunter K.B., Rice K.R., Ward D.S., Trost S.G. (2012). Factors associated with physical activity in children attending family child care homes. Prev. Med..

[b0110] Harris P.A., Taylor R., Thielke R., Payne J., Gonzalez N., Conde J.G. (2009). Research electronic data capture (REDCap)--a metadata-driven methodology and workflow process for providing translational research informatics support. J. Biomed. Inf..

[b0115] Harte S., Theobald M., Trost S.G. (2019). Culture and community: observation of mealtime enactment in early childhood education and care settings. Int. J. Behav. Nutr. Phys. Act..

[b0120] He M., Tucker P., Gilliland J., Irwin J.D., Larsen K., Hess P. (2012). The influence of local food environments on adolescents’ food purchasing behaviors. Int. J. Environ. Res. Public Health.

[b0125] Head Start Program Performance Standards and Other Regulations Washington, D.C.: U.S. Dept. of Health and Human Services, Administration for Children and Families, Head Start Bureau.; 2000. Available from: https://purl.fdlp.gov/GPO/LPS53786.

[b0130] Hubbs-Tait L., Mulugeta A., Bogale A., Kennedy T.S., Baker E.R., Stoecker B.J. (2009). Main and interaction effects of iron, zinc, lead, and parenting on children’s cognitive outcomes. Dev. Neuropsychol..

[b0135] Hughes C.C., Gooze R.A., Finkelstein D.M., Whitaker R.C. (2010). Barriers to obesity prevention in Head Start. Health Aff. (Millwood).

[b0140] Kunin-Batson A.S., Seburg E.M., Crain A.L., Jaka M.M., Langer S.L., Levy R.L. (2015). Household factors, family behavior patterns, and adherence to dietary and physical activity guidelines among children at risk for obesity. J. Nutr. Educ. Behav..

[b0145] Laughlin L. Who’s Minding the Kids? Child Care Arrangements: Spring 2011 census.gov United States Census Bureau 2013 [cited 2015 24 August ]. Available from: http://www.census.gov/prod/2013pubs/p70-135.pdf.

[b0150] Lazarus M.A., Tandon P.S., Otten J.J. (2018). Examining relationships between food procurement characteristics and nutritional quality in washington state child care settings. Childhood Obesity.

[b0155] Lin B.H., Morrison R.M. (2002). Higher fruit consumption linked with lower body mass index. FoodReview.

[b0160] Lindsay A.C., Salkeld J.A., Greaney M.L., Sands F.D. (2015). Latino family childcare providers’ beliefs, attitudes, and practices related to promotion of healthy behaviors among preschool children: a qualitative study. J. Obesity.

[b0165] Lorson B.A., Melgar-Quinonez H.R., Taylor C.A. (2009). Correlates of fruit and vegetable intakes in US children. J. Am. Diet. Assoc..

[b0170] Lynch M., Batal M. (2011). Factors influencing childcare providers’ food and mealtime decisions: an ecological approach. Child Care Pract..

[b0175] Mikkila V., Rasanen L., Raitakari O.T., Pietinen P., Viikari J. (2005). Consistent dietary patterns identified from childhood to adulthood: the cardiovascular risk in Young Finns Study. Br. J. Nutr..

[b0180] Mikkila V., Rasanen L., Raitakari O.T., Pietinen P., Viikari J. (2004). Longitudinal changes in diet from childhood into adulthood with respect to risk of cardiovascular diseases: the Cardiovascular Risk in Young Finns Study. Eur. J. Clin. Nutr..

[b0185] Monsivais P., Kirkpatrick S., Johnson D.B. (2011). More nutritious food is served in child-care homes receiving higher federal food subsidies. J. Am. Diet. Assoc..

[b0190] Moore L.V., Diez Roux A.V. (2006). Associations of neighborhood characteristics with the location and type of food stores. Am. J. Public Health.

[b0195] Perkins C. (2004 July). Knowing your purveyors keeps food quality high. Nation’s Restaurant News..

[b0200] 13. Dietary Recommendations for Healthy Children: American Heart Association; 2018 [cited 2020 July 14]. Available from: https://www.heart.org/en/healthy-living/healthy-eating/eat-smart/nutrition-basics/dietary-recommendations-for-healthy-children.

[b0205] Redford, J., Desrochers, D., Hoyer, K.M. 2017. The Years before School: Children’s Nonparental Care Arrangements from 2001 to 2012. Stats in Brief. NCES 2017-096. National Center for Education Statistics.

[b0210] Rivera J.A., Hotz C., Gonzalez-Cossio T., Neufeld L., Garcia-Guerra A. (2003). The effect of micronutrient deficiencies on child growth: a review of results from community-based supplementation trials. J. Nutr..

[b0215] Rule A.C., Stewart R.A. (2002). Effects of practical life materials on kindergartners’ fine motor skills. Early Childhood Educ. J..

[b0220] Schwartz M.B., Henderson K.E., Grode G., Hyary M., Kenney E.L., O'Connell M. (2015). Comparing current practice to recommendations for the child and adult care food program. Childhood Obesity.

[b0225] Shariff Z.M., Bond J.T., Johnson N.E. (2000). Nutrition and educational achievement of urban primary schoolchildren in Malaysia. Asia Pac. J. Clin. Nutr..

[b0230] Sisson S.B., Kiger A.C., Anundson K.C., Rasbold A.H., Krampe M., Campbell J.E. (2017). differences in dietary intake of young children at childcare and home. Prevent. Med. Rep..

[b0235] Tovar A., Risica P., Mena N., Lawson E., Ankoma A., Gans K.M. (2015). An assessment of nutrition practices and attitudes in family child-care homes: implications for policy implementation. Prevent. Chronic Dis..

[b0240] Tovar A., Mena N.Z., Risica P., Gorham G., Gans K.M. (2015). Nutrition and physical activity environments of home-based child care: what hispanic providers have to say. Childhood Obesity.

[b0245] US Dept of Agriculture. Child and Adult Care Food Program 2017 [cited 2017 February]. Available from: http://www.fns.usda.gov/cnd/care/.

[b0250] USDA Economic Research Service. Rural-Urban Commuting Area Codes 2016 [cited March 19 2019]. Available from: https://www.ers.usda.gov/data-products/rural-urban-commuting-area-codes/.

[b0255] USDA. Child and Adult Care Food Program: United States Department of Agriculture, Food and Nutriton Service; 2018 [cited 2019 January]. Available from: http://www.fns.usda.gov/cacfp/meals-and-snacks.

[b0260] USDA. ChooseMyPlate 2019 [cited 2019 July 2019]. Available from: https://www.choosemyplate.gov/how-much-does-my-preschooler-need.

[b0265] USDA. Healthy Hunger-Free Kids Act: United States Department of Agriculture, Food and Nutriton Service; 2017 [cited 2018 July]. Available from: https://www.fns.usda.gov/school-meals/healthy-hunger-free-kids-act.

[b0270] Whitaker R.C., Gooze R.A., Hughes C.C., Finkelstein D.M. (2009). A national survey of obesity prevention practices in Head Start. Arch. Pediatr. Med..

[b0275] Williams J., Scarborough P., Matthews A., Cowburn G., Foster C., Roberts N. (2014). A systematic review of the influence of the retail food environment around schools on obesity-related outcomes. Obes. Rev..

[b0280] Williams B.D., Sisson S.B., Dev D.A., Lowery B., Horm D., Campbell J. (2021). Associations between Community built environments with early care and education classroom physical activity practices and barriers. Int. J. Environ. Res. Public Health.

[b0285] Zaltz D.A., Pate R.R., O'Neill J.R., Neelon B., Benjamin-Neelon S.E. (2018). Barriers and facilitators to compliance with a state healthy eating policy in early care and education centers. Child Obes..

[b0290] Zaltz D.A., Hecht A.A., Neff R.A., Pate R.R., Neelon B., O'Neill J.R. (2020). Healthy eating policy improves children’s diet quality in early care and education in South Carolina. Nutrients.

