# Supplementary material for: Relationships between proximity to grocery stores and Oklahoma Early Care and Education classroom nutrition practices
Source: Prev Med Rep. 2022 Jul 21;29:101917. doi: 10.1016/j.pmedr.2022.101917 (PMC9352449; doi:10.1016/j.pmedr.2022.101917)
Supplement: Supplementary Data 1 [file mmc1.docx]

| Supplementary Table 1. Food purchasing methods, miles to purchasing and nearest grocery stores, and percent urban/rural among Oklahoma ECE programs participating in the Communities and Classroom Health Survey in 2019-2020, by GIS-determined proximity to grocery stores (*n*=373). | | | | | |
| --- | --- | --- | --- | --- | --- |
|  | Low Proximity (*n*=115) | | Access to Grocery (*n*=258) | |  |
|  | % or mean | *SD* | % or mean | *SD* | *p*-value |
| Methods for Purchasing Center Foods (%) |  |  |  |  | **<0.0001*** |
| In-person shopping at a store | 48.2 | - | 50.1 | - | - |
| Online ordered then picked up in person | 21.5 | - | 15.9 | - | - |
| Online and delivered | 15.5 | - | 22.1 | - | - |
| Over the phone with a vendor | 14.6 | - | 11.6 | - | - |
| Roundtrip Miles to Purchasing Center Foods (mean, *SD*) | 20.5 | 20.7 | 15.5 | 22.1 | **0.0067*** |
| Distance in Miles to Nearest Grocery Store (mean%, *SD*) | 3.9 | 4.3 | 1.0 | 1.4 | **0.0138*** |
| Percent Urban/Rural within Census Tract (%) |  |  |  |  | **<0.0001*** |
| Urban | 88.0 | - | 45.9 | - | - |
| Rural | 11.9 | - | 54.0 | - | - |
| ECE= center for Early Childhood Education. GIS= Geographic Information Systems.  Wilcoxon Rank Sum test and Fisher’s Exact test were used to determine differences in ECE contextual demographic characteristics between those located within low proximity areas versus those within accessible proximity of grocery store. ***indicates significant difference among groups (*p*-value<0.05).** | | | | | |
